# Transcriptional splice variants of CD40 and its prognostic value in breast cancer

**DOI:** 10.3906/biy-1912-21

**Published:** 2020-04-02

**Authors:** Neşe ÜNVER, Diğdem YÖYEN ERMİŞ, Bahar Zehra WEBER, Güneş ESENDAĞLI

**Affiliations:** 1 Department of Stem Cell Sciences, Graduate School of Health Sciences, Center for Stem Cell Research and Development,Hacettepe University, Ankara Turkey; 2 Department of Medical Biology, Faculty of Medicine, Lokman Hekim University, Ankara Turkey; 3 Institute of Cancer Research, Medical University of Vienna, Vienna Austria; 4 Department of Basic Oncology, Cancer Institute, Hacettepe University, Ankara Turkey

**Keywords:** CD4, breast cancer, alternative splicing, transcriptional variant, antitumor response

## Abstract

CD40 is an important tumor necrosis factor receptor (TNFR) family protein for the development of antitumor response against cancer cells, apart from its role in the regulation of the immune system as a costimulatory molecule. It is broadly expressed on the surface of immune cells and in diverse cancer types, including breast cancer. Here, we analyzed both CD40/CD40 ligand expression in breast cancer cells and tissues using public data sets and overall survival analysis in ungrouped breast cancer patients, as well as in the triple-negative breast cancer subtype. We detected CD40 gene expression along with its 3 different splice variants (variants 1–3), predominantly in the triple-negative subgroup of breast cancer cell lines. The results of the overall survival analysis showed that high CD40 gene expression, particularly in the triple-negative subgroup of breast cancer patients, is associated with better survival. In addition to the transcriptional levels of CD40 splice variants, investigation of protein levels of these variants will allow the categorization of breast cancer cells and reveal their potential as an immunotherapeutic target.

## 1. Introduction

CD40, a member of the tumor necrosis factor (TNF) family, is a 48 kDa Type I transmembrane glycoprotein. It is expressed on the surface of B cells, macrophages,and dendritic cells, as well as in various cancer cell lines and tissues including non-small cell lung cancer, ovarian, urinary bladder cancer, breast melanoma, pancreatic cancer, colon cancer, and B-lineage malignancies (u1d3b; u1d3e).

The interaction between CD40 and its ligand, CD40 ligand (CD40LG), orchestrates dendritic cell–T cell crosstalk, dendritic cell tolerance, and conversion of CD8+T cell exhaustion (Ara et al., 2018). Agonistic antibodies against CD40 display T cell dependent antitumor activity, especially when used in combination with chemotherapy and immune checkpoint inhibitors (Bonaventura et al., 2019).

CD40 expression is induced by lipopolysaccharides (LPS)at the transcriptional level via nuclear factor kappa B (NF-κB) and signal transducer and activator of transcription 1 alpha (STAT-1α) in macrophages and microglia (Qin et al., 2005). Its expression is regulated at the posttranscriptional level through alternative splicing when different variants are formed. Only 3 of these variants (variants 1, 2, and 3) can be converted to a functional CD40 protein. The CD40 Type 1 variant is the longest isoform, whereas the CD40 Type 2 variant is the soluble form of the protein. Type 3 variant lacking the cysteine-rich subunit-3/4 (CRD-3 and CRD-4) display low ligand-binding affinity (Tone et al., 2001).

CD40–CD40LG interaction stimulates growth inhibition in ovarian, bladder, ovarian, squamous epithelial carcinoma, and cervical cancer cell lines (Tong et al., 2001). Agonistic anti-CD40 antibodies either directly activate macrophages, dendritic cells, or B cells, or indirectly activate them through cytotoxic lymphocytes and natural killer cells (Baxendale et al., 2005). CD40 pathway activation is linked with an improved pathologic response to preoperative trastuzumab-plus-T/FEC chemotherapy in breast tumors (Bonaventura et al., 2019). It has also been suggested that IL-2/CD40 or IL-15/CD40 combination therapy may be more effective than anti-CD40 alone for the advanced treatment of solid tumors, including renal cell carcinoma (Piechutta and Berghoff, 2019).

Ligation of the trimeric recombinant protein (rCD40LG) with CD40 leads to growth inhibition of breast cancer cells (Slobodova et al., 2011;Gomes et al., 2009). Moreover, it has been shown that cytoplasmic expression of CD40 is correlated with a better prognosis in breast cancer (Slobodova et al., 2011). Apart from CD40 expression, membrane CD40LG expression has been rarely detected in tumor-infiltrating lymphocytes in breast cancer tissues, supporting their low capacity to inhibit growth of breast cancer cells via the CD40–CD40LG axis (Tong et al., 2001). In contrast, its expression is correlated with an unfavorable patient prognosis in soft tissue sarcomas (Baxendale et al., 2005; Ottaiano et al., 2004).CD40 has been associated with a poor prognosis for stages 3 and 4 esophageal squamous cell carcinoma patients as well (Matsumura et al., 2016).

In this study, we showed the transcriptional variants of the CD40 gene in different breast cancer cell lines. We also aimed to analyze its prognostic value and expression with its cognate ligand molecule, CD40 ligand (CD40LG),via public databases.

## 2. Materials and methods

Cell culture:BT-474, HCC-38, MCF-7, MDA-MB-231, MDA-MB-468, SK-BR-3, T-47D, ZR-75-1 breast cancer cell lines and MCF-12A healthy mammary epithelial cell line were cultured in RPMI medium containing L-glutamine (2 mM), 10% fetal bovine serum (Biological Industries, Cromwell, CT, USA), penicillin (100 unit/mL) and streptomycin (100 mg/mL; Biochrom, Cambridge, UK).Cell culture was also conducted in the presence of LPS (1 ug/mL, 24h), which is known to induce CD40 expression. 

RNA isolation and Reverse transcription-polymerase chain reaction (RT-PCR):Total RNA was isolated from cells using RNeasy Mini Kit (QIAGEN, Hilden, Germany).The RNA samples were treated with DNA-free kit (Ambion, Waltham, MA, USA). Following RNA isolation, cDNA was synthesized using RevertAidTM First Strand cDNA Synthesis Kit (Fermentas, Waltham, MA, USA). Primers specific for CD40 variants were designed and synthesized by Iontek (İstanbul, Turkey). We used the following primer sequences (from 5′ to 3′) for the PCR: Variant 1 human CD40 forward: tgcccagtcggcttcttct, reverse: AGTCTTGTTTGTGCCTGCCTG;Variant 2 human CD40 forward:TTGGACAAGGTCCCCAGGA, reverse: GGGGCCTTATTGGTTGGCT; Variant 3 human CD40 forward: AGCAGATTGGTCCCCAGGA, reverse:GGGGCCTTATTGGTTGGCT. CD40 variant 1–3 expression was investigated using the RT-PCR method.PCR was initiated by one hold of 95 °C for 10 min, followed by 35 cycles of 15 s at 95 °C, 55 s at 60 °C for V2 and V3 or 55 s at 61 °C for V1, and 5 s at 72 °C, followed by one hold of 72 °C for 10 min. b-actin was used as an internal control. RT-PCR products were separated using 2% agarose gel electrophoresis.

Analysis of CD40/CD40LG gene expression from the CCLE and TCGA databases: The RNA-seq expression data of the CD40 gene from all of the cancer cell lines were downloaded and a total of 66 breast cancer cell lines were selected. To check the correlation between CD40 and its ligand CD40LG, The Cancer Genome Atlas (TCGA, Provisional) data obtained from 529 patients with breast invasive carcinoma were evaluated.

Survival analysis: CD40 and its cognate receptor CD40L Gwere evaluated with Kaplan–Meier plotter tool (http://kmplot.com/analysis/index.php)(Gyorffy et al., 2010). Patient cohorts were divided into high and low expression groups according to quantile expression of the CD40 and CD40LG genes.Hazard ratios (HR) with 95% confidence intervals and log rank P value were calculated.

Statistical analysis: Significant correlations between CD40 and CD40LG gene expression levels were evaluated using the Pearson correlation. Statistical significance was indicated using a P value of 0.05 or less based on a two-tailed test. Analyses were performed using GraphPad Prism version 8.1.1 (GraphPad, San Diego, CA, USA).

## 3. Results

### 3.1. High CD40 expression is associated with a good prognosis in breast cancer

**Figure 1:**
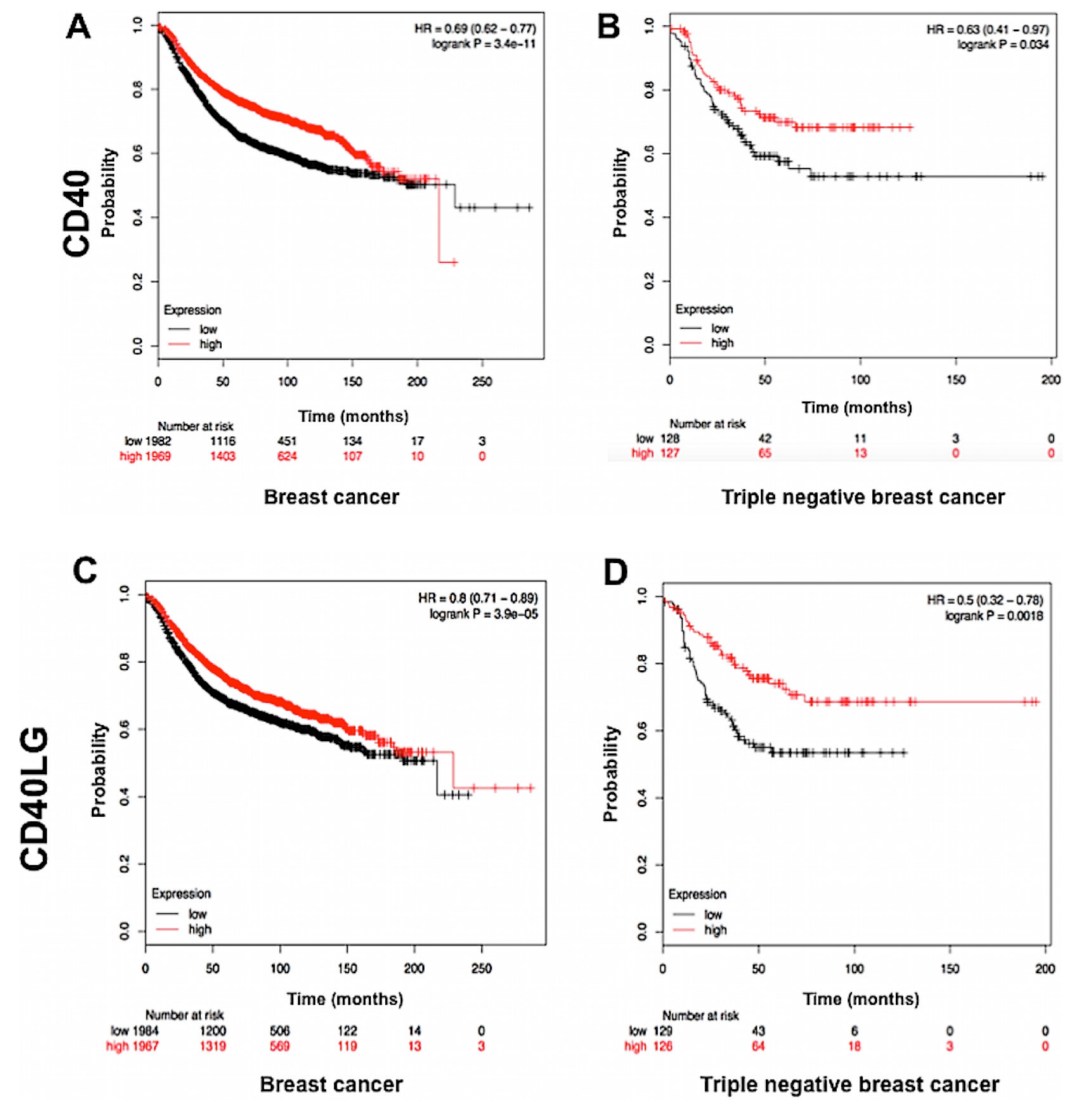
Prognostic value of CD40 mRNAexpression determined using the Kaplan–Meier plotter (A–B). a) Survival curves for all patients with breast cancer (n = 3951). b) Survival curves for patients with triple negative breast cancer (n = 255). HR: Hazard ratio. Prognostic value of CD40LG mRNA expression determined using the Kaplan–Meier plotter (C–D). c) Survival curves for all patients with breast cancer (n=3951). D) Survival curves for patients with triple negative breast cancer (n=255).

Prognostic value of CD40 gene expression was determined in breast cancer using the Kaplan–Meier plotter. In 3951 patients with breast cancer, survival analyses showed that high CD40 mRNA expression was highly associated with improved overall survival (OS) (hazard ratios of 0.62–0.77, P =3.4e-11) (Figure 1a). Furthermore, overall survival was better in the high-expression group vs. the low-expression group in patients with triple-negative breast cancer, which is the most aggressive subtype (hazard ratios of 0.41–0.97, P =0.034) (Figure 1b). Likewise, high CD40LG expression was associated with better survival in all breast cancer (hazard ratios of 0.71–0.89, P = 3.9e-05) and triple negative breast cancer (hazard ratios of 0.32–0.78, P = 0.0018) data set (Figure 1c and Figure 1d).

### 3.2. Breast cancer cell lines express CD40 at mRNA levels 

**Figure 2:**
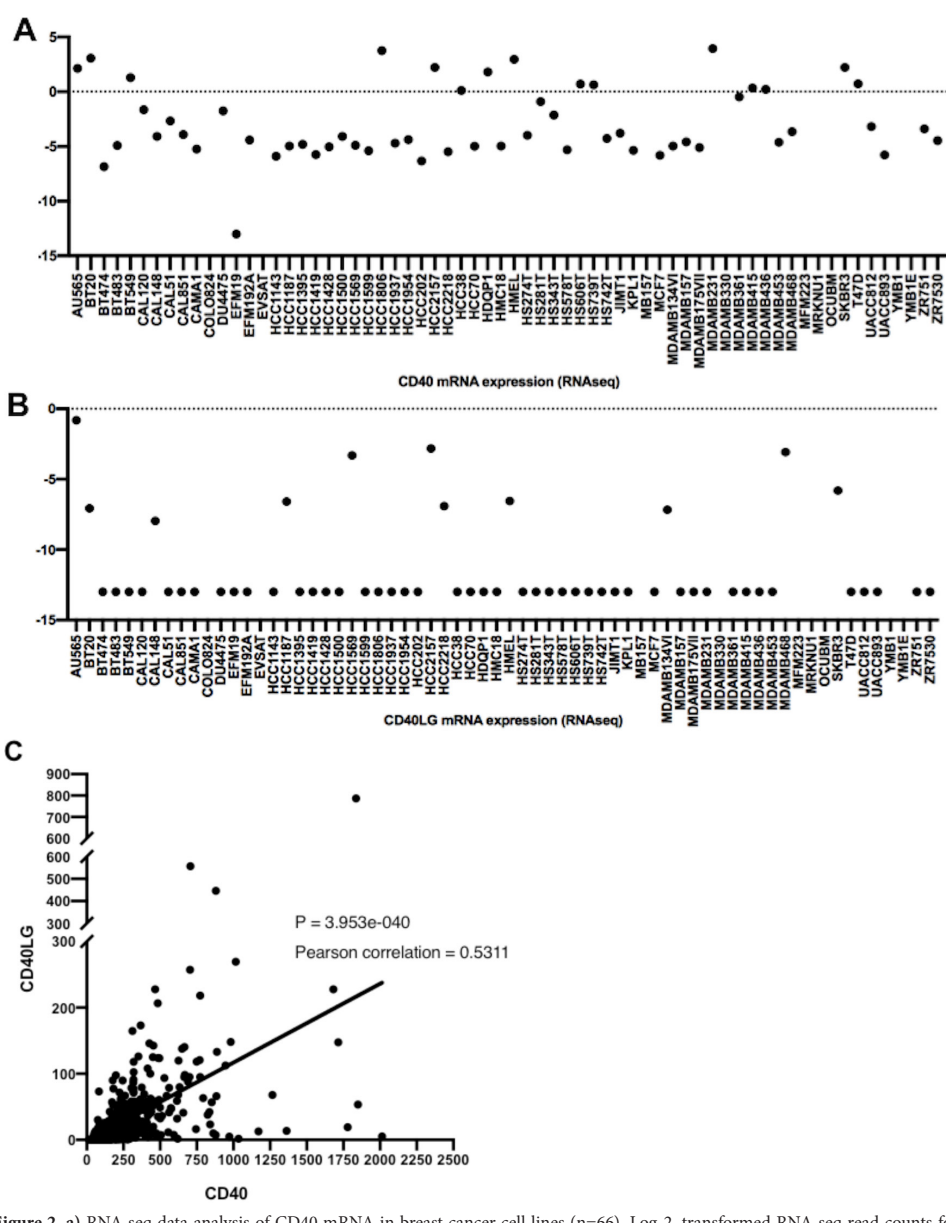
a) RNA-seq data analysis of CD40 mRNA in breast cancer cell lines (n=66). Log-2–transformed RNA-seq read counts for CD40 gene are shown on the y-axis. b) RNA-seq data analysis of CD40LG mRNA in breast cancer cell lines (n=66). Log-2–transformed RNA-seq read counts for CD40LG are shown on the y-axis. c) Correlation between CD40 and CD40LG gene expression based on the TCGA breast cancer data set.

Gene expression data for cancer cell lines were downloaded from the Cancer Cell Line Encyclopedia (CCLE) database (Barretina et al., 2012) at www.broadinstitute.org/ccle. RNA-seq analysis of 66 human breast cancer cell lines derived from the CCLE data setshowed that breast cancer cells display low CD40 expression (Figure 2a). Data analysis of these breast cancer cell lines revealed extremely low CD40LG expression as well (Figure 2b).

On the other hand, it has been shown that CD40 is significantly correlated with CD40LG gene expression in 529 patients with invasive carcinoma of the breast according to the TCGA (Provisional) data set of the Agilent microarray platform (Pearson correlation: 0.53, P < 0.05) (Figure 2c).

### 3.3. Demonstration of CD40 gene expression with its splice variants

We checked the 3 isoforms, which can be converted to protein (Figure 3A). CD40 exons that correspond to protein domains including cysteine-rich domain (CRD) 1–4 are shown in Figure 3b. Three specific primers were designed to detect splice variants V1–3 (Figure 3b). Specific PCR amplicons were detected by agarose gel electrophoresis and β-actin was used as an internal control (Figure 3c).

**Figure 3.1:**
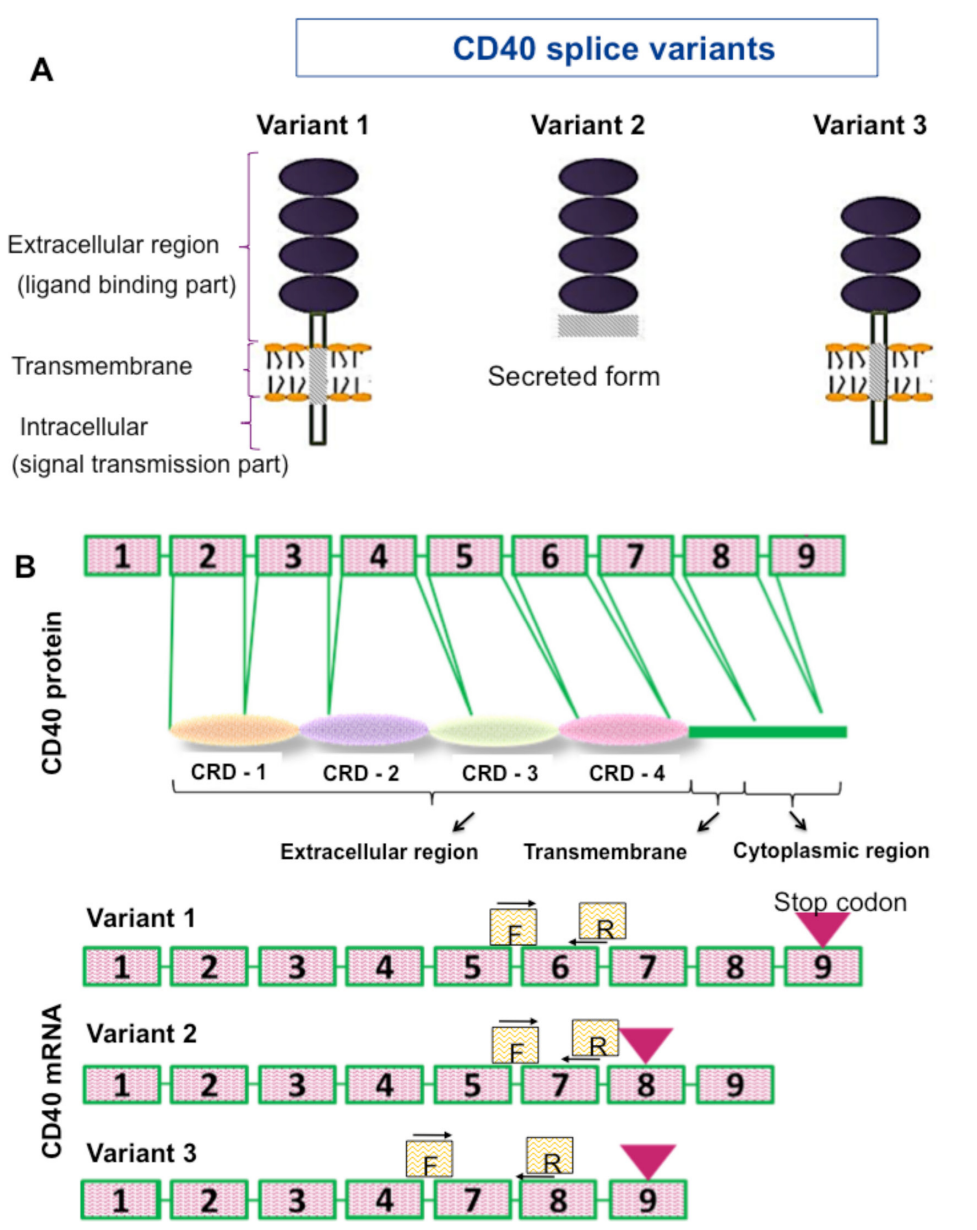
a) Schematic demonstrations of CD40 splice variants. b)Illustration of exon regions of CD40 splice variants indicated by boxes, exon–intron sites to which primers designed specifically binds, and CD40 protein domains corresponding to exon regions. c) Agarose gel images regarding amplicons of CD40 variants (V1=115 bp, V2=130 bp, V3=130 bp). d) Expression of CD40 splice variants in breast cancer cell lines cultured with or without LPS. C (–) represents negative PCR control; C(+) represents positive PCR control.

**Figure 3.2:**
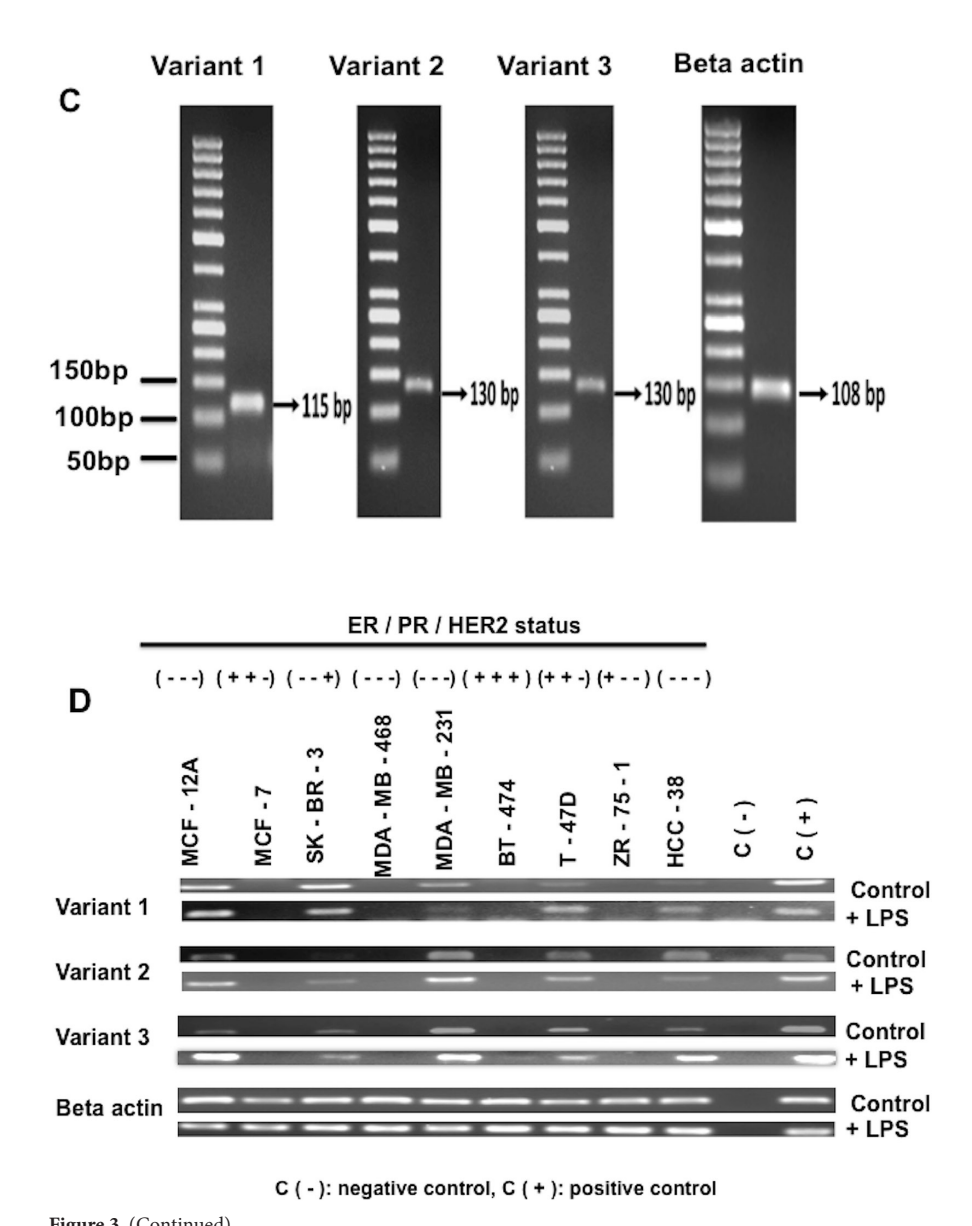
(Continued).

In order to show expression of CD40 splice variants, the levels of gene expressions were examined using primers designed specific for the variants. Six out of 8 breast cancer cell lines expressed CD40 variant/variants. There was no expression detected in MCF-7, MDA-MB-468, and ZR-75-1. All3 CD40 variants were detected in cell culture conditions with and without LPS in SK-BR-3, MDA-MB-231, T-47D, and HCC-38 cell lines. Similarly, MCF-12A normal breast cells showed a similar transcriptional pattern as these breast cancer cells for CD40 splice variants. In the BT-474 cell line, CD40 variant 3 was only detected after induction with LPS in cell culture. In the absence of LPS, BT-474 cells were CD40-negative (Figure 3d).

## 4. Discussion 

CD40, one of the best characterized costimulatory molecules, has contributed to cell-mediated and humoral immune responses in the immune system(Loskog and Eliopoulos, 2009). In addition to its function on the immune system, coexpression of CD40 and its ligand CD40L mediates oncogenic effects on immortalized human epithelial cells in vitro (Baxendale et al., 2005). Early phase trials regarding CD154 gene transfer have shown various cellular consequences, including cytokine production and enhanced Fas-mediated tumor apoptosis, following CD40 ligation(Weiss et al., 2009;Tong and Stone, 2003). 

CD40 expression exists on the basal part of stratified squamous epithelium, suggesting its modulatory role in cell growth and differentiation (Young et al., 1998).Epithelial CD40 is limited to self-renewing stem cells. In murine fibroblasts, constitutive CD40 signaling plays a pivotal role in the transformation of the cells (Esteva et al., 2007). It has also been detected in different types of carcinoma cells. However, its ligand CD40LG has barely been detected in normal and malignant epithelial cells (Tong et al., 2001; Tong and Stone, 2003). CD40 has been detected in all compartments, namely membrane, cytoplasm, and nucleus, in breast cancer (u1d45; u1d46). Additionally, Kaplan–Meier progression-free survival curves for non-small cell lung cancer patients with malignant pleural effusion showed that high soluble CD40 is associated with poor prognosis (HR = 0.4643, P = 0.045 (Mu et al., 2015). Since soluble CD40 expression would suppress CD40LG+ T cells and contribute to immune escape mechanisms (Kim et al., 2015), it is critical to explore this splice variant in cancer cells.

Overall, the survival analysis that we performed, in contrast to the studies on solid tumors mentioned earlier, has shown that high CD40 expression is associated with good prognosis in triple-negative breast cancer. Similarly, tumor-associated CD40 expression has a favorable prognostic effect in renal cell carcinoma and diffuse large B-cell lymphoma following chemotherapy (Weiss et al., 2014;Rydstrom et al., 2010). CD40 expression is accompanied by CD8 T cell infiltration in renal cell carcinoma, indicating that it may be useful as a biomarker for patient survival (Weiss et al., 2014). Both the detection of CD40 expression in triple-negative cells and the association of high CD40 expression with good prognosis led us to clarify variant expressions of this gene. All CD40 splice variants (V1–3) were detected in 3 out of 4 triple-negative breast cancer cell lines. It appears that CD40 expression and its stimulation in cancer cells could be dependent on the cancer cell type-specific signaling pathway and the immune niche within the cancer microenvironment.

In this study, we aimed to show CD40 splice variants under normal conditions, as well as in the presence of LPS, a strong transcriptional inducer of CD40 gene (Dong et al., 2008). Previous experiments performed on peripheral blood monocytic cells showed that LPS induction augments CD40 mRNA and protein. LPS causing IκBα degradation leads to NF-κB activation. Furthermore, the activation of NF-κB has been shown to be essential for CD40 expression stimulated with LPS on human monocytes, as determined in murine macrophages and microglia (Pearson et al., 2001; Wu et al., 2009).

As a result of our experimental assays, we detected the expression of all 3 variants (V1, V2, and V3) in MCF-12A, MDA-MB-231, SK-BR-3, T-47D, and HCC-38, which are ER–/PR–cell lines, except T-47D, which is classified as Luminal A (ER+PR+HER2–) (Yu et al., 2017). On the other hand, CD40 splice variants were not shown in MCF-7, MDA-MB-468, and ZR-75-1 cell lines which diverged from each other. In the earlier study, consistent with our results, BT-20 was also identified as CD40-positive, while MCF-7 and ZR-75-1 were CD40-negative (Esteva et al., 2007). Treatment with CD40 ligand decreased proliferation of BT-20 and T-47D cells which are CD40-positive, but did not change the growth of CD40-negative breast cancer cell lines, as detected in MCF-7 or ZR-75-1 cells (Esteva et al., 2007), supporting the idea that CD40–CD40LG axis modulates proliferation of cancer cells. Interestingly, the ER+/PR+/HER2+ breast cancer cell line BT-474 does express Variant 3 in the presence of LPS. High CD40 expression was detected in the cytoplasm of hormone-receptor–positive breast cancer, whereas it was detected on the surface membrane of triple-negative breast cancer (Kim et al., 2015; Slobodova et al., 2011). We thought that the basal expression of V3, which has low ligand binding capacity, might be quite low in BT-474 and could be stimulated by LPS.

When the demonstration of CD40 variant expressions is technically considered, it can be mentioned that not showing the variants by sequencing and not quantifying the expression differences of CD40 variants using quantitative real-time PCR is among the handicaps of the experimental assay.Therefore, no further quantitative evaluation of gene expression has been performed due to the absence of CD40 expression in some of the breast cancer cell lines and for the purpose of displaying the existence of CD40 variants (V1–3) only.

In conclusion, investigation of CD40 splice variants’ expression among different types of breast cancer cells is important for targeting the secreted or full-length form of CD40 mRNA. Development of antibodies specific for CD40 splice variants may provide insight into molecular mechanisms regarding the CD40 signaling pathway. Additionally, histopathological analysis of breast tumor biospecimens in terms of different splice variants of CD40 would be informative for determination of potential targets and discrimination between the soluble and full-length forms of the protein. Whether or not CD40 variant expression change occurs after the use of agonistic CD40 antibodies should be taken into consideration. Further analysis of the genetic structure and functionality of CD40 and CD40LG would allow the development of more useful prognostic factors and prognostic prediction tools in breast cancer subtypes. 

## Acknowledgments

This work did not receive any specific grant from funding agencies.
